# Evaluating the New York City Emergency Department Syndromic Surveillance for Monitoring Influenza Activity during the 2009-10 Influenza Season

**DOI:** 10.1371/500563f3ea181

**Published:** 2012-08-17

**Authors:** Emily Westheimer, Marc Paladini, Sharon Balter, Don Weiss, Anne Fine, Trang Quyen Nguyen

## Abstract

Objective: To use laboratory data to assess the specificity of syndromes used by the New York City emergency department (ED) syndromic surveillance system to monitor influenza activity. Design: For the period from October 1, 2009 through March 31, 2010, we examined the correlation between citywide ED syndrome assignment and laboratory-confirmed influenza and respiratory syncytial virus (RSV). In addition, ED syndromic data from five select NYC hospitals were matched at the patient and visit level to corresponding laboratory reports of influenza and RSV. The matched dataset was used to evaluate syndrome assignment by disease and to calculate the sensitivity and specificity of the influenza-like illness (ILI) syndrome. Results: Citywide ED visits for ILI correlated well with influenza laboratory diagnoses (R=0.92). From October 1, 2009, through March 31, 2010, there were 264,532 ED visits at the five select hospitals, from which the NYC Department of Health and Mental Hygiene (DOHMH) received confirmatory laboratory reports of 655 unique cases of influenza and 1348 cases of RSV. The ED visit of most (56%) influenza cases had been categorized in the fever/flu syndrome; only 15% were labeled ILI. Compared to other influenza-related syndromes, ILI had the lowest sensitivity (15%) but the highest specificity (90%) for laboratory-confirmed influenza. Sensitivity and specificity varied by age group and influenza activity level. Conclusions: The ILI syndrome in the NYC ED syndromic surveillance system served as a specific but not sensitive indicator for influenza during the 2009-2010 influenza season. Despite its limited sensitivity, the ILI syndrome can be more informative for tracking influenza trends than the fever/flu or respiratory syndromes because it is less likely to capture cases of other respiratory viruses. However, ED ILI among specific age groups should be interpreted alongside laboratory surveillance data. ILI remains a valuable tool for monitoring influenza activity and trends as it facilitates comparisons nationally and across jurisdictions and is easily communicated to the public.

## Background

Many local and state public health departments have come to rely on syndromic surveillance to monitor and characterize large-scale, seasonal disease trends and have reported it to be especially informative for tracking influenza trends,^1^ ^2^ ^3^ despite limited (if any) ability for laboratory-based validation at the individual level. Regularly monitoring disease trends through syndromic surveillance is of importance to jurisdictions of all sizes as it enables public health practitioners to detect and monitor the onset and trajectory of community-wide disease circulation. Syndromic surveillance of influenza also allows for description of age, geographic and other epidemiologic features of a given outbreak that could inform interventions such as recommending vaccination and other countermeasures, and providing data to providers and hospitals regarding trends in patient visits.

The categorization into syndromes of electronic data captured at the time of a health-related encounter (e.g., hospital emergency department (ED) chief complaints, pharmacy medication sales) is the basis of both the simplicity and lack of specificity of syndromic surveillance. Syndrome assignment occurs outside of the health encounter system, most often performed by an automated algorithm at local and state health departments. Syndromic surveillance systems offer a snapshot of care-seeking patterns but do not represent diagnoses, as the syndromes are neither clinically nor laboratory confirmed at the time of analysis. For example, a patient presenting to the ED in New York City with a complaint of fever and cough would be categorized into the influenza-like illness (ILI) syndrome, without information about laboratory test results.^1^ Routine analysis of syndromic surveillance data is usually conducted by health department analysts the day after health-related encounters to provide a timely picture of disease-related trends that could inform prevention or testing messages, as well as identify possible anomalies of public health concern such as a bioterrorism attack or localized outbreak.

The NYC Department of Health and Mental Hygiene (DOHMH) developed a chief complaint-based emergency department syndromic surveillance system in 2001. Not until the 2009-10 influenza season (as part of enhanced surveillance established after the initial wave of pandemic 2009 H1N1) did DOHMH have the opportunity to evaluate the validity of its influenza-related syndromes. The system regularly monitors six major syndromes, several of which have been shown to correlate well with expected seasonal respiratory and diarrheal disease trends.^2^ Two syndromes, respiratory, which includes chief complaints of cough, shortness of breath and upper respiratory infection, and fever-flu, defined as fever, viral syndrome, chills/body aches, or flu, have been used in NYC to monitor influenza trends; in 2008 the ILI syndrome definition was added. ILI is limited to chief complaints of fever with sore throat, fever with cough, or flu/influenza. Although the volume of ILI syndrome visits is much lower than of respiratory or fever-flu visits, several characteristics supported the use of ILI for monitoring influenza activity in NYC^2^ :

· Synchrony of the ILI definition with the Centers for Disease Control and Prevention ILI definition.^4^

· High ecological correlation between citywide ILI syndrome and influenza isolate trends.^2^

· Low baseline ILI levels during non-epidemic periods.

· Simplicity of the definition for communicating information to the public. The respiratory, fever-flu, and ILI syndromes are all monitored, but only ILI syndrome trend data are shared with the public during influenza season.

Because of concerns about a second wave of 2009 H1N1 influenza during the 2009-2010 influenza season, NYC DOHMH selected 5 sentinel hospitals for surveillance of the epidemiology and severity of hospitalized influenza-positive cases in NYC. The DOHMH linked patient ED visits with their laboratory influenza and RSV testing results in order to evaluate the correlation, sensitivity and specificity of the influenza-related syndromes. We share the methods and findings to offer guidance to other jurisdictions that might consider evaluating their syndromes, as well as to describe the implications of monitoring ILI in ED surveillance.

## Methods

We conducted two main analyses of NYC ED syndromic data and laboratory-confirmed data captured during the 2009-10 influenza season: (1) a citywide, non-matched correlation analysis, and (2) a patient-specific matched analysis of syndromes and laboratory test results from the five sentinel hospitals.

**Data Sources.** *NYC Syndromic and Disease Surveillance.* The DOHMH syndromic surveillance system receives daily ED visit data from 49 out of 54 NYC hospitals, which includes an estimated 95% of citywide ED visits. The DOHMH received approximately 4,000,000 reports of ED visits in 2009. Each visit is assigned by an automated algorithm to one or more syndromes based on the patient’s chief complaint.^5^ The respiratory, fever/flu, and ILI syndromes are defined above. For the purposes of the evaluation described here, visits could be categorized as one or more of these syndromes.

Laboratory-positive influenza and RSV of all NYC residents are reportable to DOHMH. Electronic reports of positive influenza and RSV tests from laboratories are sent via the New York State Electronic Clinical Laboratory Reporting System (ECLRS). In 2008 there were 1436 distinct case reports of influenza; however, in 2009 (because of 2009 H1N1 influenza), 14,523 cases were reported. Laboratory-positive RSV was made reportable in NYC in 2008; in 2009 more than 5,000 cases were reported.

*NYC Sentinel Surveillance for Influenza, Fall/Winter 2009-10.* Five hospitals, one in each county of NYC, were selected for enhanced sentinel surveillance for hospitalized influenza cases during the 2009-2010 influenza pandemic. Hospitals were selected based on volume of visits, their participation in the ED syndromic surveillance system, and the presence of both adult and pediatric emergency departments. A dedicated nurse at each facility located patients admitted to inpatient units with fever and respiratory symptoms and tested them for influenza. Because of lack of weekend coverage and variable patient load, identifying all patients was not possible. Specimens were collected by nasopharyngeal swab and transported on ice in standard viral media to the NYC Public Health Laboratory (PHL). Specimens not transported within 48 hours were frozen at -70C and transported to the PHL on dry ice. Specimens were tested using the CDC Influenza Virus Real-time RT-PCR Detection and Characterization Panel. PHL reported positive results via ELCRS and negative influenza results directly to DOHMH. A subset of specimens negative for influenza was also tested for RSV.

*Matching of Sentinel Hospital Laboratory Reports to Emergency Department Visits.* Sentinel hospitals included patient medical record numbers (MRN) in both the ED daily electronic data file and all laboratory reports (sent to ECLRS) for inpatients and outpatients, allowing linkage of ED visits to corresponding positive laboratory test results for influenza or RSV. For those patients who had multiple ED visits during the period of interest, only an ED visit three days before or after the date of the laboratory test date was matched to the laboratory report. When there were multiple ED visits within the three-day window, the visit closest to the date of disease diagnosis on a matching lab report was used. Figure 1 shows the flow of patient information from all data sources.

**Flowchart of citywide and sentinel hospital data of both syndromic emergency department visits and laboratory testing results for influenza and RSV, New York City, October 1, 2009 - March 31, 2010:**
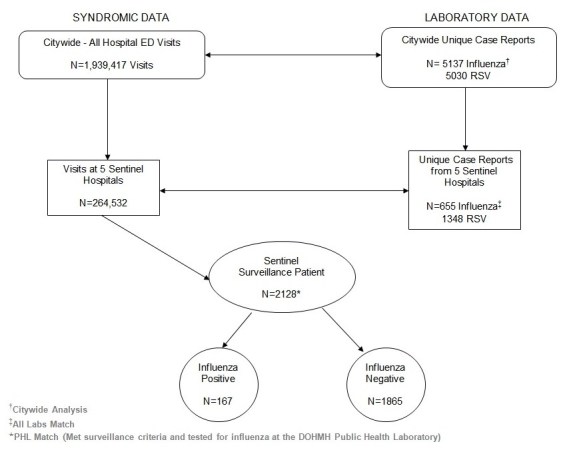

**Analyses.** Data were analyzed by age group: 0-4, 5-17, 18-64, 65+ years, and all ages. The 0-4 age group was of particular interest because of its high use of emergency departments and the higher prevalence in this age group of non-influenza pathogens that can cause ILI, especially RSV.

*Citywide: Correlation of Citywide ED Syndromes and Laboratory Diagnoses (unmatched data).* For the period from October 1, 2009, through March 31, 2010, all daily ED visits from the 49 syndromic hospitals and any electronic positive influenza and RSV reports from across NYC were analyzed. To examine the daily citywide correlation in trends, we calculated the correlation between visit volume for the 3 influenza-related ED syndrome and laboratory-confirmed influenza and RSV, using ED visit date and laboratory diagnosis date. Correlation coefficients were calculated using Microsoft Excel.

*All Labs Match — All ED Patients Seen and Tested at the 5 Sentinel Hospitals: Syndrome Assignment by Laboratory Diagnosis (matched data).* Using the matched dataset of ED visits and positive ECLRS tests, we evaluated the assignment of syndromes to each case of RSV or influenza reported from one of the sentinel hospitals. Patients included in this analysis had to have been evaluated in an ED, as well as have tested positive for influenza or RSV as reported by the hospital’s laboratory.

*PHL Match — Qualifying Patients Admitted to the 5 Sentinel Hospitals: Sensitivity and Specificity of ED Syndromes and Laboratory Diagnoses (matched data).* A subset of patients analyzed in the All Labs Match could be used to calculate the sensitivity and specificity of the fever-flu, respiratory and ILI syndromes for influenza because PHL testing provided negative results. Sensitivity of each syndrome was calculated as the proportion of lab-confirmed influenza cases where the corresponding ED visit was correctly classified as ILI, fever/flu, or respiratory syndrome accordingly. PHL-confirmed influenza results served as the gold standard.

**Human Subjects.** All data included in this analysis were generated as part of routine public health surveillance and not human subjects research.

## Results

**Citywide and Sentinel Hospital ED Visits and Laboratory Testing.** From October 1, 2009 through March 31, 2010, there were 1,939,417 ED visits reported through the syndromic surveillance system, of which 264,532 (13.6%) were from the five sentinel hospitals (Table 1). Citywide, 34% (52,083) of the fever/flu syndrome visits and 18% (35,989) of the respiratory syndrome visits were also assigned to the ILI syndrome.

**Table 1. Number and percentage of emergency department (ED) visits and the corresponding syndromes at 5 Sentinel Hospitals, New York City, October 1, 2009-March 31, 2010.** **ED Syndrome** (syndromes are not mutually exclusive) **N** (% of total)

	Total ED visits	ILI	Fever/Flu	Respiratory
All Hospitals	1,939,417	52,359 (2.7)	154,339 (8.0)	203,717 (10.5)
5 Sentinel Hospitals	264,532 (13.6)	4,996 (1.9)	24,100 (9.1)	29,859 (11.3)

During the same period, we received confirmatory laboratory reports of 5137 unique cases of influenza and 5030 cases of RSV citywide. The correlation coefficients between the syndromes and positive influenza or RSV results were all high and not appreciably different, as reflected when they are displayed on the same graph with shared scales (Figure 2). Influenza across all ages correlated best with ILI (0.92) and fever/flu (0.91) followed by respiratory (0.84), whereas RSV correlated best with fever/flu (0.82), followed closely by ILI (0.78) and respiratory (0.77) syndromes. Despite similar correlation coefficients throughout the influenza season, the ILI syndrome more closely mirrored the trajectory of influenza cases when the number of cases spiked at the beginning of the season (Figure 3). Even though among the 0-4 age group influenza was equally correlated with ILI and respiratory syndromes (0.92), whereas RSV correlated best with the fever/flu syndrome (0.90) (Figure 4), influenza was driven primarily by ages 5 and over (Figure 5).

**Weekly citywide emergency department visits for (a) ILI syndrome, (b) Resp (respiratory) syndrome, (c) FevFlu (fever/flu) syndrome vs. citywide laboratory reports for influenza and RSV, New York City, October 1, 2009-March 31, 2010, all ages:**
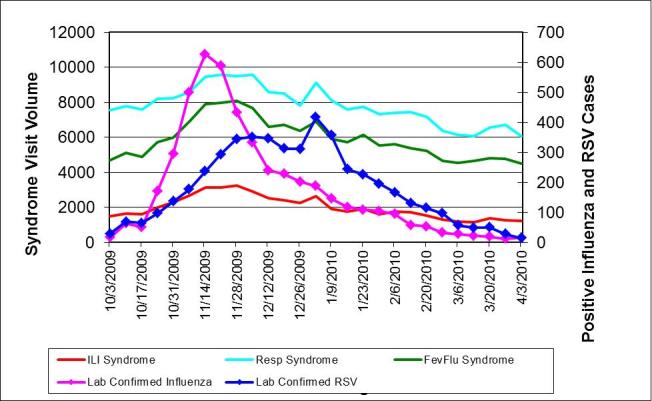

**Weekly citywide emergency department visits for (a) ILI syndrome, (b)Respiratory syndrome, (c) Fever/Flu syndrome vs. citywide laboratory reports for influenza and RSV, New York City, October 1, 2009-March 31, 2010, all ages:**
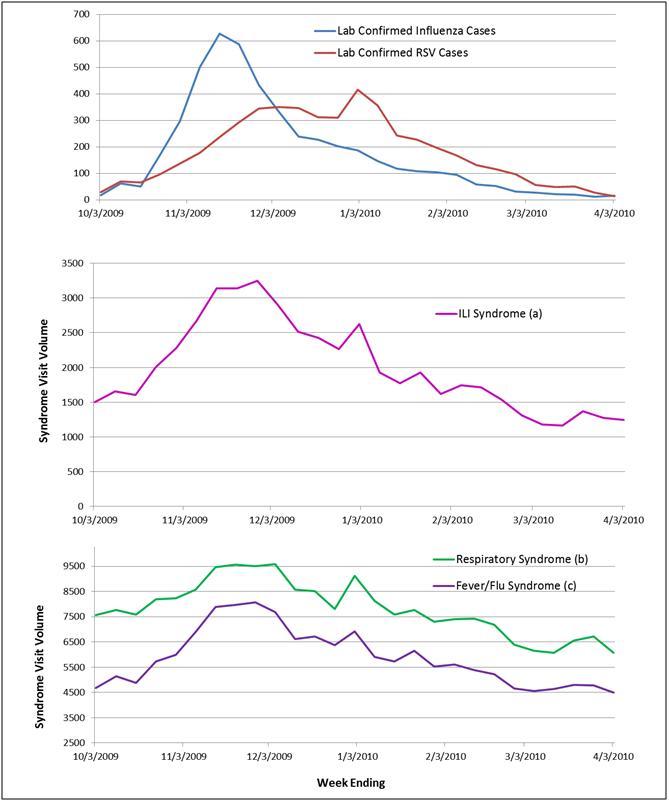

**Weekly citywide emergency department visits for (a) ILI syndrome, (b) Resp (respiratory) syndrome, (c) FevFlu (fever/flu) syndrome vs. citywide laboratory reports for influenza and RSV, New York City, October 1, 2009-March 31, 2010, 0 to 4 year-old age group:**
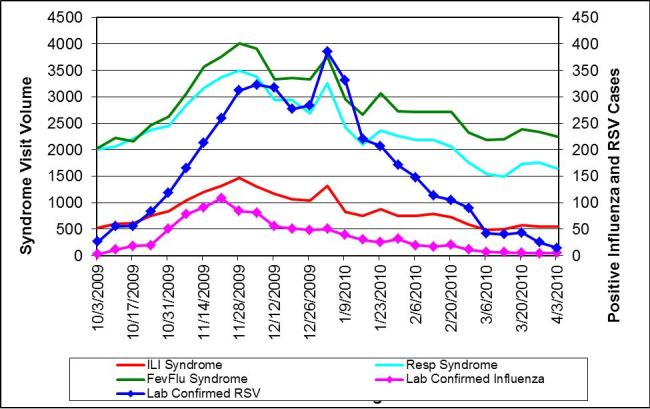

**Weekly citywide emergency department visits for ILI syndrome vs. citywide laboratory reports for influenza and RSV, New York City, October 1, 2009-March 31, 2010, by age group:**
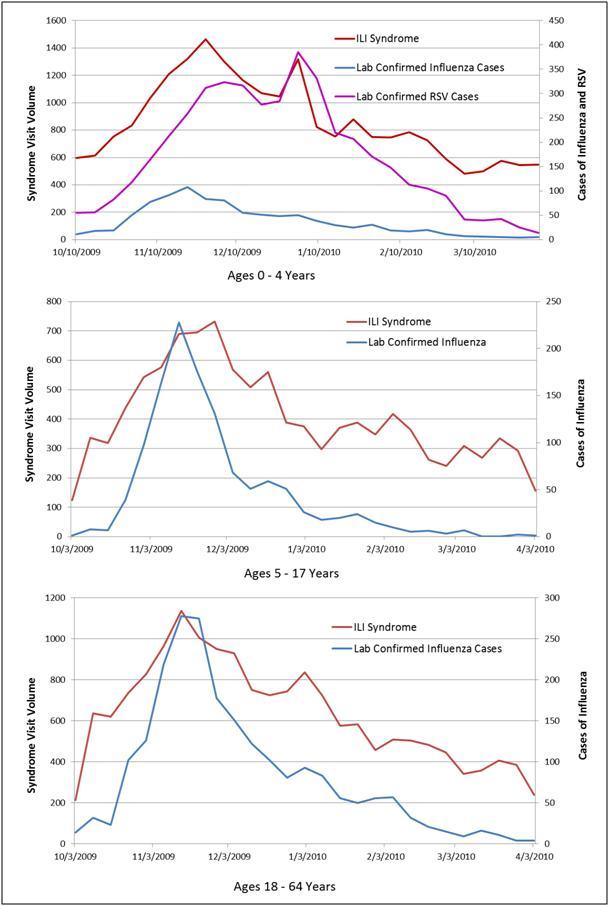

**All Labs Match: Matching ED Visit Syndrome to Positive Laboratory Report.** During the study period, the five sentinel hospitals reported 264,532 ED visits, 655 influenza cases and 1348 RSV cases. Overall, 71% of influenza and 75% of RSV reports were matched to an ED visit. Of note, more than 50% of the influenza and RSV reports came from one hospital, which was the largest of the five sentinel hospitals.

At the time of their ED visit, the majority of influenza cases had been categorized in the fever/flu syndrome with only 15% assigned to ILI (Table 2). In total there were 179 ED visits with matching lab reports classified as ILI; 109 (61%) were RSV reports and 70 (39%) were influenza.

**Table 2. Number of influenza and RSV cases and the corresponding syndromes at 5 Sentinel Hospitals, New York City, October 1, 2009-March 31, 2010.** **ED Syndrome** (syndromes are not mutually exclusive) **N** (% of total) **‡** row percentages of total matched

Lab-confirmed Sentinel Cases	Lab-Confirmed Sentinel Cases Matched to ED Visit	ILI^‡^	Fever/Flu^‡^	Respiratory^‡^	Other^‡^
Influenza (N=655)	466	70 (15)	260 (56)	165 (35)	102 (22)
RSV (N=1348)	1017	109 (11)	426 (42)	539 (53)	187 (18)

**PHL Match: Sensitivity and Specificity of Syndromes.** Of the 2128 admitted patients tested for influenza as part of the sentinel hospital surveillance, 1992 patients’ ED visits could be matched by medical record number to their influenza test result. Using these 1992 matches, the sensitivity and specificity were calculated for each syndrome. Most unmatched cases were patients who were directly admitted to the hospital by their provider and therefore did not have an ED encounter.

Most of the 1992 matches had been categorized as respiratory (47.6%), fever/flu (33.0%) or ILI (9.4%) syndrome. Only 197 (9.9%) of the matched ED visits were categorized in a non-flu-related syndrome; review of these syndromes revealed no particular pattern. There were 167 influenza A positive cases among the 1992 matched cases. The ILI syndrome had the lowest sensitivity but the highest specificity for laboratory confirmed influenza (Figure 6).

**Sensitivity and Specificity of emergency department chief complaint syndromes for laboratory confirmed influenza overall and by age group and month of diagnosis, 5 sentinel hospitals, New York City, October 1, 2009 March 31, 2010.**
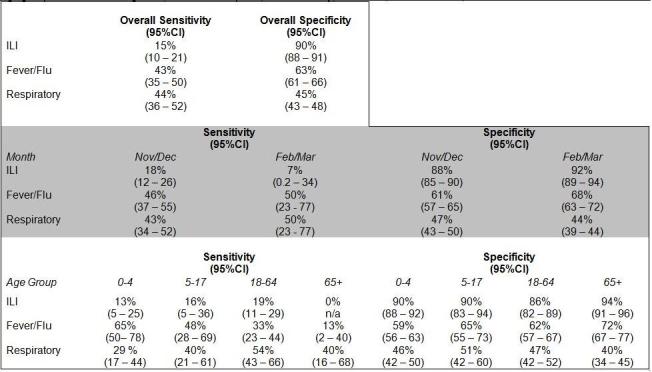

Data were further analyzed by month of diagnosis and age (Figure 6). There were no significant differences in trends by county (data not shown). Visits to the ED earlier in the influenza season (November and December) when citywide influenza activity was highest revealed a greater sensitivity and lower specificity of ILI than visits later in the season (February and March) when influenza activity was lower. By age group, ILI was a more sensitive indicator for influenza among the 5-17 and 18-64 age groups. Across syndromes, though, the respiratory syndrome was most sensitive among 18-64 year-olds, whereas fever/flu was a more sensitive measure among 0-4 year-olds. Of note, in the 65 and over age group, ILI was not assigned to any of the 15 patients in our sample who tested positive for Influenza A, resulting in a 0% sensitivity of ILI in this age group. A larger sample would be required to determine a more precise estimate.

## Discussion

The ILI syndrome in the NYC ED syndromic surveillance system served as a specific but not sensitive indicator for influenza during the 2009-2010 influenza season. While the ILI syndrome provided a more precise estimate of the initiation and trends of influenza activity, the syndrome greatly underestimated the magnitude of illness. ILI was most sensitive in the 5-17 and 18-64 age groups. Influenza illness among patients 65 and over was better represented by the respiratory syndrome, whereas patients aged 0-4 were better represented by the fever/flu syndrome. Overall, the sensitivity of ILI was higher in the months of high influenza activity (November and December 2009) as compared to the months of lower activity (February and March 2010).

Despite its limited sensitivity, the ILI syndrome might be more informative for monitoring overall trends in influenza activity because it is less likely to capture cases of other respiratory viruses. Because citywide influenza surveillance usually focuses on activity at the population level, with additional interest in specific age groups or local subgroups (e.g., county), syndromic ED data can provide useful tracking information on the trajectory of an outbreak. Among age subgroups, though, ILI in children 0 to 4 years should be interpreted with caution and alongside laboratory data as both the literature and our data show that the ILI syndrome in this age group reflects both RSV and influenza activity.^6^ ^7^ In the over 65 age subgroup, influenza cases were more likely assigned the respiratory syndrome, reflecting their greater likelihood of presenting to EDs without mention of fever or with an underlying respiratory disease (possibly worsened by influenza infection) as part of their chief complaint. 8 However, it is unclear whether the improved specificity of ILI over other syndromes would have an appreciable impact from a public health perspective with regard to informing the public and providers about the start of influenza season or guidelines with regard to testing and treatment.

There are several limitations to this analysis. First, the majority of all laboratory reports of influenza came from a single sentinel hospital. The overrepresentation of this hospital might have skewed the analysis of sensitivity and specificity among the matches between ED visits and a laboratory diagnosis, which may not accurately reflect citywide trends. The sensitivity and specificity of ILI for four of the five hospitals were within the 95% confidence interval range of the overall ILI results presented in Figure 6, with the exception being one of the smallest hospitals, so the one hospital overrepresented in the sample did not seem to influence these results. In addition, because hospital EDs differ in their methods (e.g., free-text chief complaint vs. pull-down menu) and idiomatic expressions when coding chief complaint, this high-volume hospital could over-represent different illness patterns as compared to the other four hospitals. In addition, a limited number of hospitals in the city perform testing for RSV; one of these hospitals was included in the five sentinel hospitals, therefore weighting the RSV results towards this hospital’s ED practices and patient population. In general, the five sentinel EDs might not be representative of citywide EDs or the NYC population.

Second, the analyses were conducted on data captured during the 2009-10 influenza season during the H1N1 pandemic, an atypical influenza season in terms of the distribution of disease by age group, symptom presentation, and timing of peak activity. Different influenza seasons produce more or less extreme trends depending on circulating strains, susceptible populations and health care access behavior. The season described in this manuscript is unique in that it followed the massive off-season 2009 H1N1 pandemic, which likely resulted in a larger immune population for the regular 2009-10 influenza season. Furthermore there could have been a change in healthcare utilization patterns during the fall/winter as a result of the pandemic outbreak during the spring and summer of 2009. Because syndromic surveillance can only monitor healthcare-seeking behavior of those who visit an emergency room, changes in syndrome patterns from season to season may reflect changes in healthcare behaviors and not necessarily changes in illness spread or severity. Therefore, this analysis may not be generalizable to other influenza seasons when other influenza strains may be circulating. In addition, we were comparing the syndromic data only to influenza and RSV reports and did not account for other respiratory viruses that were likely circulating during the same period, such as rhinovirus. The sensitivity and specificity analysis also has limited generalizability because this analysis was conducted only with data from patients admitted to the hospital rather than both inpatients and outpatients.

Finally, these results are subject to testing biases as well as variability in emergency department utilization by age and socio-economic status. During times of high influenza activity, providers may opt not to test patients for influenza because of the need to make immediate treatment decisions. These untested ED patients may or may not be captured as ILI but would not have been included in this analysis because of the lack of testing. In seasons of relatively mild influenza activity, emergency department activity might be reduced, therefore limiting the ability to monitor ILI trends. Furthermore, because RSV is a disease predominantly recognized in young children, older children and adults are infrequently tested for RSV; similarly, children aged 0-4 years may not be tested for influenza as frequently as they are tested for RSV. Therefore, the true prevalence of RSV in older children and adults and the true prevalence of influenza in young children might be biased by testing behavior. In our matched analysis, 41% of the influenza results and 95% of the RSV results come from the 0-4 year age category. In contrast, the 0-4 year age group made up only 12% of the total ED visit volume during this period and 2.5% of patients admitted to the hospital.

## Conclusion

This analysis of matched laboratory test results for specific pathogens with specific ED syndromes has shown that use of the ILI syndrome as a marker for trends in influenza activity is valid in NYC. Syndromic surveillance of ILI, while not a useful method alone for identifying all cases, can provide trend data that can be used to inform public health messages and clinical practice — once ILI activity has risen above a pre-determined threshold, clinicians can be certain to include influenza in their differential diagnoses of patients with flu-like symptoms. As another example, among the 0-4 year age group, the rise of ILI and fever/flu syndromes can be monitored in relation to laboratory reports for influenza and RSV to identify the introduction and amplification of each virus each season.

From an overall public health perspective, the ILI syndrome might not provide added benefit to current surveillance activities beyond the timely identification of the beginning of influenza season. Further enhancements to syndromic surveillance could better describe trends in epidemic infections by taking into account age groups, variable hospital utilization patterns, and perhaps also different hospital practices in coding patients’ reasons for visiting the ED and in laboratory testing. In addition, the introduction of novel viruses or illnesses, or the changing epidemiology of a known seasonal illness, warrants regular monitoring of how well syndromes track with laboratory-confirmed diseases and how changes in sensitivity and specificity of syndromes might affect ongoing surveillance and the ability to estimate influenza burden, both citywide and by subgroups. Given that most laboratories around the country are already routinely reporting cases of influenza (and, in some cases, RSV), jurisdictions who already utilize syndromic surveillance could conduct a similar analysis with minimal additional input. Furthermore, as health care information technology improves, routine linkage of laboratory and syndromic data in real-time might be soon possible, which might add public health value to ongoing surveillance activities.

This analysis highlights the need to further understand hospital utilization patterns as well as hospital-specific systems and practices to better interpret syndromic data. Although the ILI syndrome may not serve as the most sensitive measure of influenza activity, ILI remains a valuable tool for monitoring influenza activity and trends as it facilitates comparisons nationally and across jurisdictions and is easily communicated to the public.
